# Supplementary material for: Plasmodium berghei liver stage parasites exploit host GABARAP proteins for TFEB activation
Source: Commun Biol. 2024 Nov 21;7:1554. doi: 10.1038/s42003-024-07242-x (PMC11582615; doi:10.1038/s42003-024-07242-x)
Supplement: Supplementary file 2 — Supplementary Information [file 42003_2024_7242_MOESM2_ESM.pdf]

Supplementary Figure S1

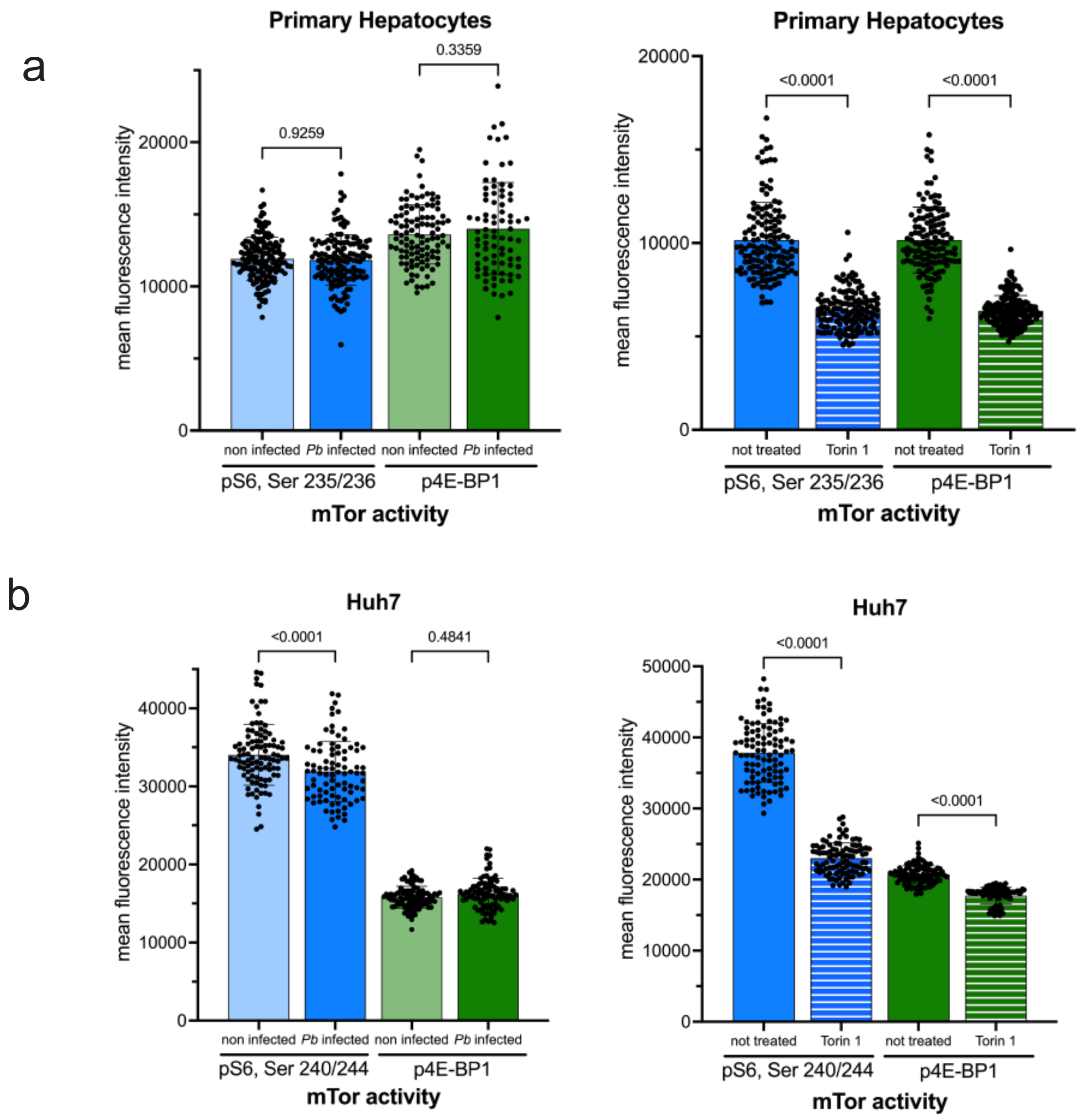

Figure S1: mTORC1 activity in *Pb*mCh infected mouse primary hepatocytes and in *Pb*mCh infected Huh7 hepatoma cells. a mTORC1 activity in *Pb*mCh infected or Torin1 treated mouse primary hepatocytes. Mouse primary hepatocytes were infected with *Pb*mCh (24 hpi) (left panel) or treated with Torin1 (200 nM, 2 h) (right panel). Cells were fixed and stained at indicated times with anti-pS6(Ser235/236) or anti-p4E-BP1 to visualize phosphorylated substrates of the mTOR kinase. Note that mTOR is active in *Pb* infected cells at the same level as in non-infected cells, and that Torin1 significantly inhibits the activity of mTOR. Pictures were taken with a widefield fluorescent microscope and fluorescence intensity was measured using Fiji. N > 80 for each sample. The graph depicts mean and SD of one representative experiment. The experiment was performed twice. P-values were calculated using a Student's t test. **b** mTORC1 activity in *Pb*mCh infected or Torin1 treated Huh7 hepatoma cells. Cells were infected with *Pb*mCh (24 hpi) (left panel) or treated with Torin1 (200 nM, 2 h) (right panel) and then fixed and stained at indicated times with anti-pS6(Ser240/244) or anti-p4E-BP1) to visualize phosphorylated substrates of the mTOR kinase. Note that mTOR is active in *P. berghei*-infected cells at the same level as in non-infected cells, and that Torin1 significantly inhibits the activity of mTOR. Pictures were taken with a widefield fluorescent microscope and fluorescence intensity was measured using Fiji. N > 60 for each sample in each experiment. The graph depicts mean and SD of the pooled data of two independent experiments. P-values were calculated using a Student's t test.

Supplementary Figure S2

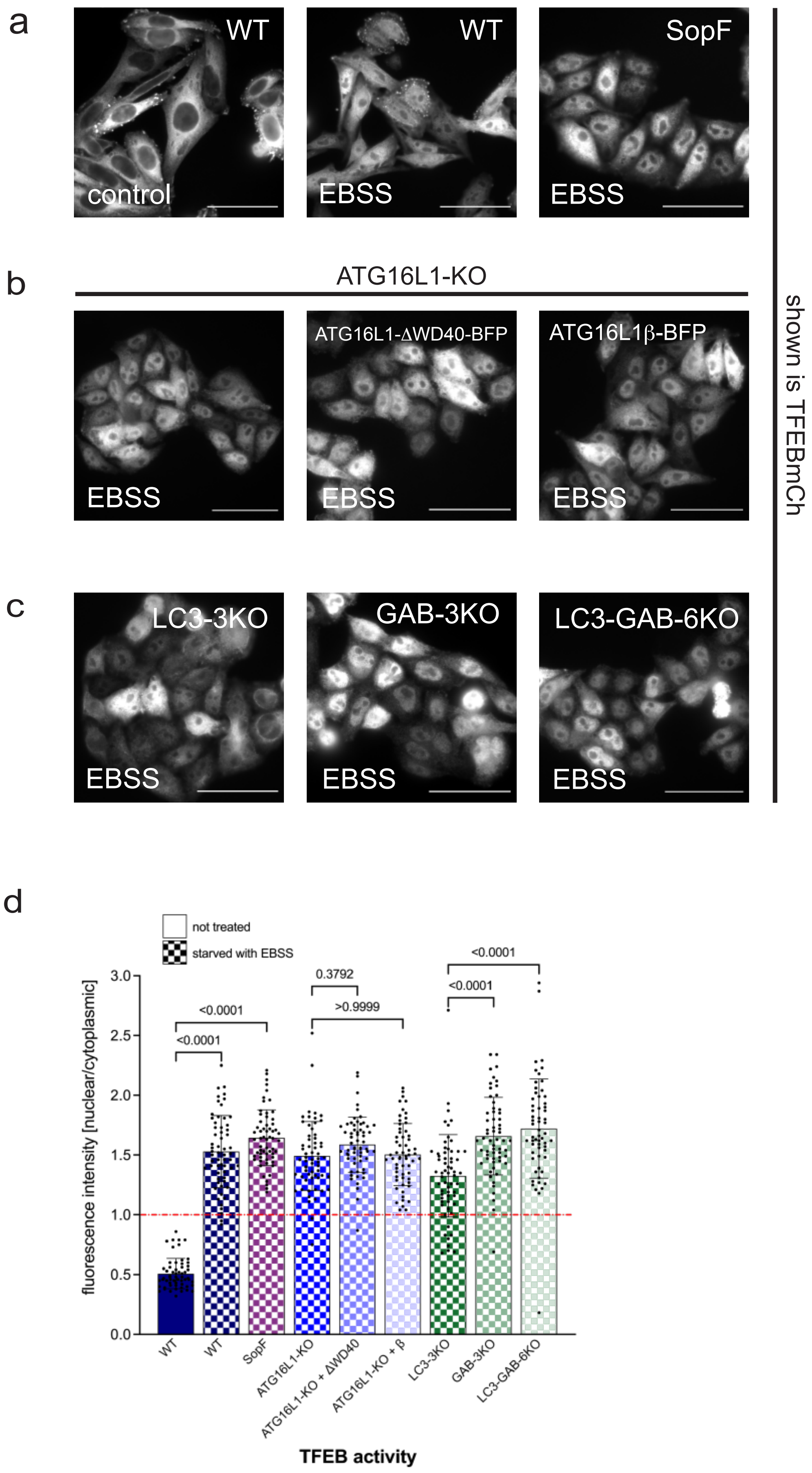

Figure S2: TFEB localization in SopF expressing cells, ATG16L1-KO and ATG8-KO cell lines upon starvation. a TFEB localization in HeLa WT and in SopF expressing cells. **b** TFEB nuclear translocation in ATG16L1-KO cells. **c** TFEB localization in different ATG8-KO cell lines. All different cell lines stably express TFEBmCh and were treated in the same way. Cells were grown on glass cover slips overnight and then starved for 2 h in EBSS. Fixed cells were stained with anti-RFP antibodies to enhance the TFEB signal (here shown in grey), pictures were taken with a widefield fluorescence microscope. Scale bar 50 µm. **d** Quantification of the experiment described in a, b, c. Cells were treated as described above. Fluorescence intensity in the nucleus and the cell cytoplasm was measured and the ratio nuclear/cytoplasmic was calculated for each cell. A ratio above 1 indicates more nuclear than cytoplasmic TFEB, a ratio lower than 1 indicates more cytoplasmic than nuclear TFEB. Pictures were analyzed using Fiji. N > 30 for each cell line in each experiment. The graph depicts mean and SD of the pooled data of two independent experiments. P-values were calculated using a one-way ANOVA test. Note that all cell lines show nuclear TFEB upon starvation.

Supplementary Figure S3

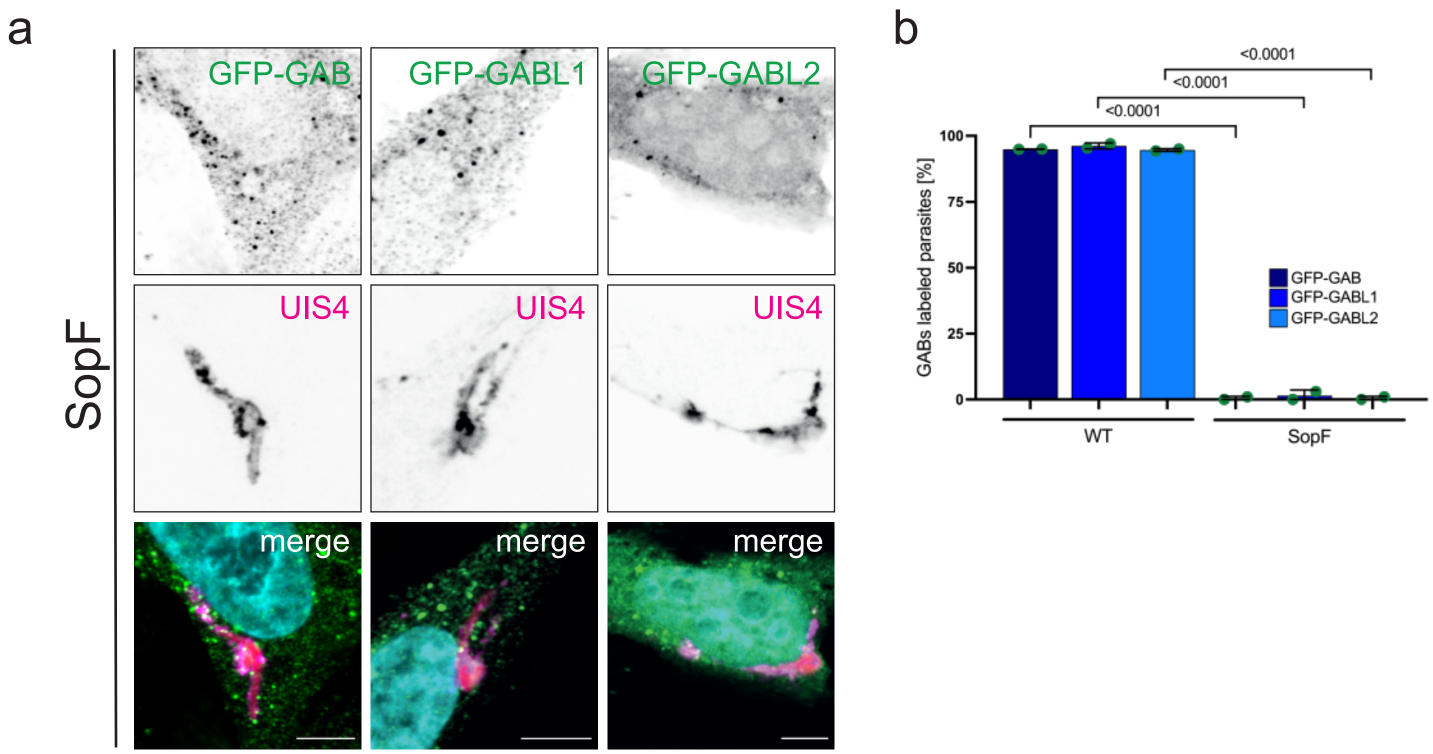

Figure S3: PVM localization of GABARAPs is inhibited by SopF. a HeLa WT cells stably expressing SopF were transiently transfected with GFP-GABARAPs and approximately 15 h post transfection infected with *Pb* mCh sporozoites. 6 hpi, infected cells were fixed and stained with anti-GFP antibodies (green) to enhance the GABARAP signal and anti-UIS4 antibodies (magenta) to visualize the *Pb* PVM. DNA was stained with Dapi (cyan). Images were taken with a confocal laser scanning microscope. Scale bar 5 µm. Note that expression of SopF inhibits the PVM localization of all GABARAPs. **b** Quantification of the experiment described in a. The graph shows the percentage of GABARAP-positive parasites in HeLa WT cells expressing SopF. Only UIS4-positive parasites were counted. The graph depicts the mean and SD of two independent experiments. P-values were calculated using a Student's t test. N ≥ 70 per experiment and cell line.

Supplementary Figure S4

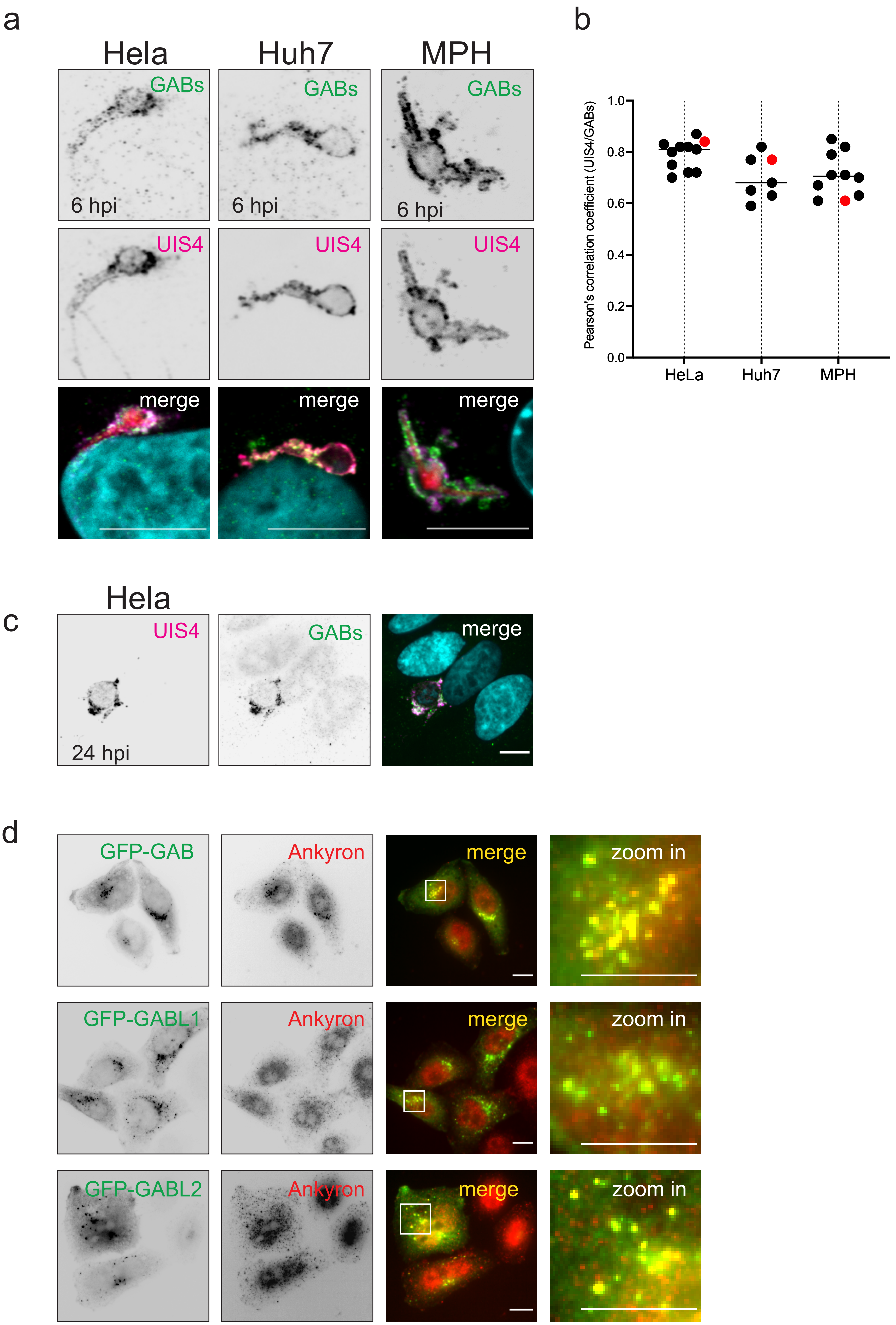

Figure S4: Endogenous GABARAPs localize to *P. berghei* PVM. a HeLa, Huh7 cells and mouse primary hepatocytes (MPH) were infected with *Pb*mCh. Infected cells were fixed 6 hpi and stained with anti-UIS4 antibodies (magenta) to visualize the *Pb* PVM and with Ankyron1191 AH5032 to visualize endogenous GABARAPs (green). DNA was stained with Dapi (cyan). Images were taken with a confocal laser scanning microscope. Scale bar 10 µm. Note that endogenous GABARAPs localize to the *Pb* PVM in all 3 cell types. **b** Quantification of the experiment described in a. Graph shows the Pearson's correlation coefficient (PCC) for UIS4 and endogenous GABARAPs. PCC was calculated using the Coloc2 tool of FIJI. N = 11 parasites for HeLa, 7 parasites for Huh7 and 10 parasites for MPH. Each dot represents one parasite, each red dot represents the parasites shown in panel a. **c** HeLa cells were infected with *Pb*mCh, fixed 24 hpi and stained with anti-UIS4 antibodies (magenta) to visualize the *Pb* PVM and with Ankyron1191 AH5032 to visualize endogenous GABARAPs (green). DNA was stained with Dapi (cyan). Images were taken with a fluorescence widefield microscope. Scale bar 10 µm. Note that GABARAPs localize to the parasite PVM 24 hpi. **d** Ankyron1191 AH5032 specifically binds to all three GABARAPs. HeLa GAB-3KO cells were transiently transfected with each of the 3 GABARAPs. 24 h after transfection cells were treated with 200 nM Torin1 for 2 h, fixed and stained with anti-GFP antibodies (green) and Ankyron1191 AH5032 (red). Pictures were taken with a fluorescence widefield microscope. Scale bar 10 µm. Note that all 3 GABARAPS were bound by the Ankyron, but the best signal is obtained with GABARAP. There is also an unspecific nuclear signal which also appears in the non-transfected cells.

Supplementary Figure S5

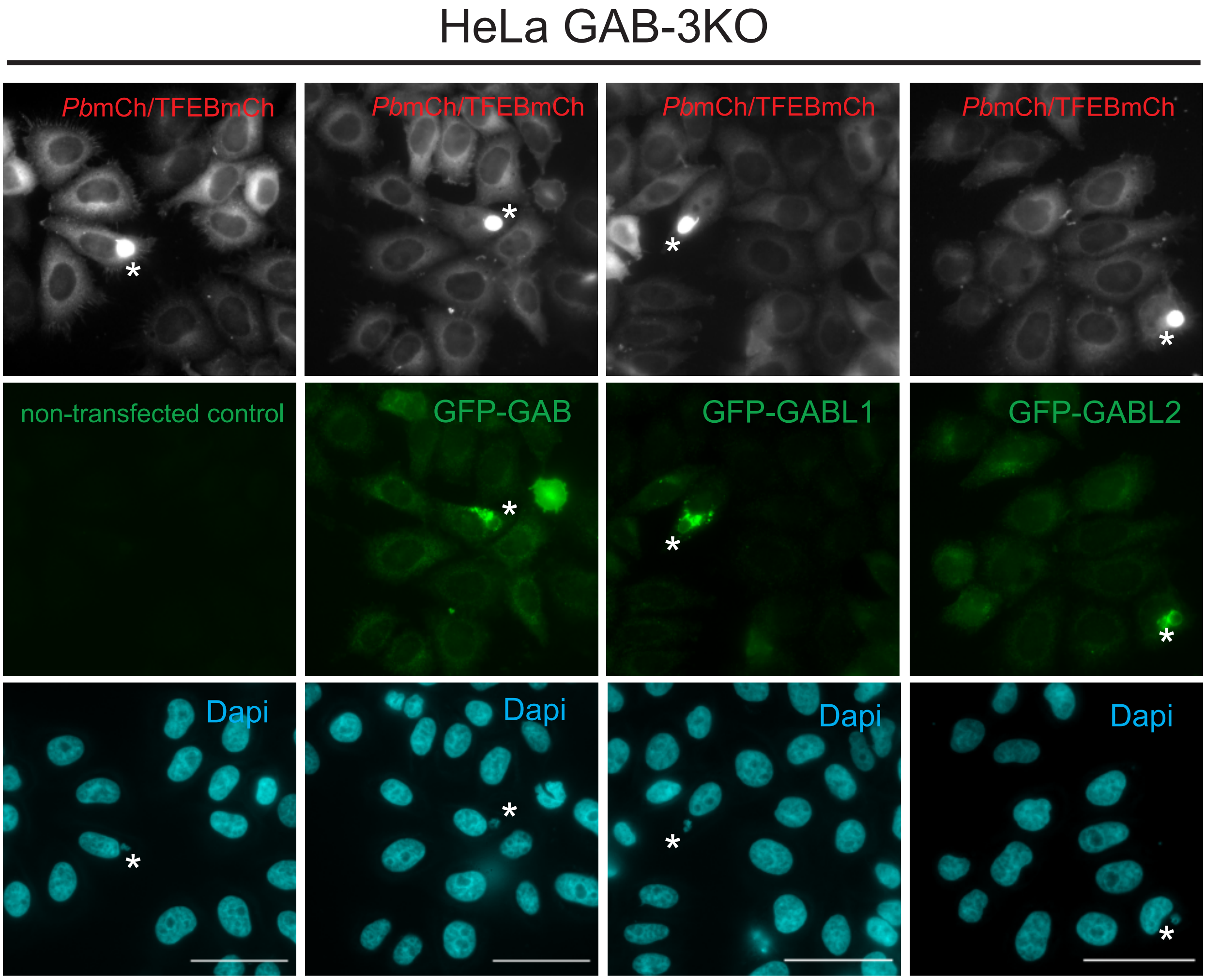

Figure S5: Expression of GFP-GABARAPs in GAB-3KO cells. IFAs used in quantification of Figure 3e. GABARAPs are needed for nuclear translocation of TFEB in *Pb* infected cells. Shown are representative pictures of non-transfected HeLa GAB-3KO cells and transiently transfected GAB-3KO cells ectopically expressing each of the GFP-GABARAPs (shown in green) all constitutively expressing TFEBmCh (shown in grey). Cells were fixed at 24 hpi and stained with anti-GFP and anti-RFP antibodies, pictures were taken with a fluorescence widefield microscope. *Pb*mCh parasites are labeled with a white asterix. Note that in GAB-3KO cells TFEB is localized in the host cell cytoplasm in *Pb* infected cells and that all GABARAPs are proficient to activate TFEB upon *Pb* infection. All GFP-GABARAPs localise to the parasite's PVM 24 hpi. Scale bar 50 µm

Supplementary Figure S6

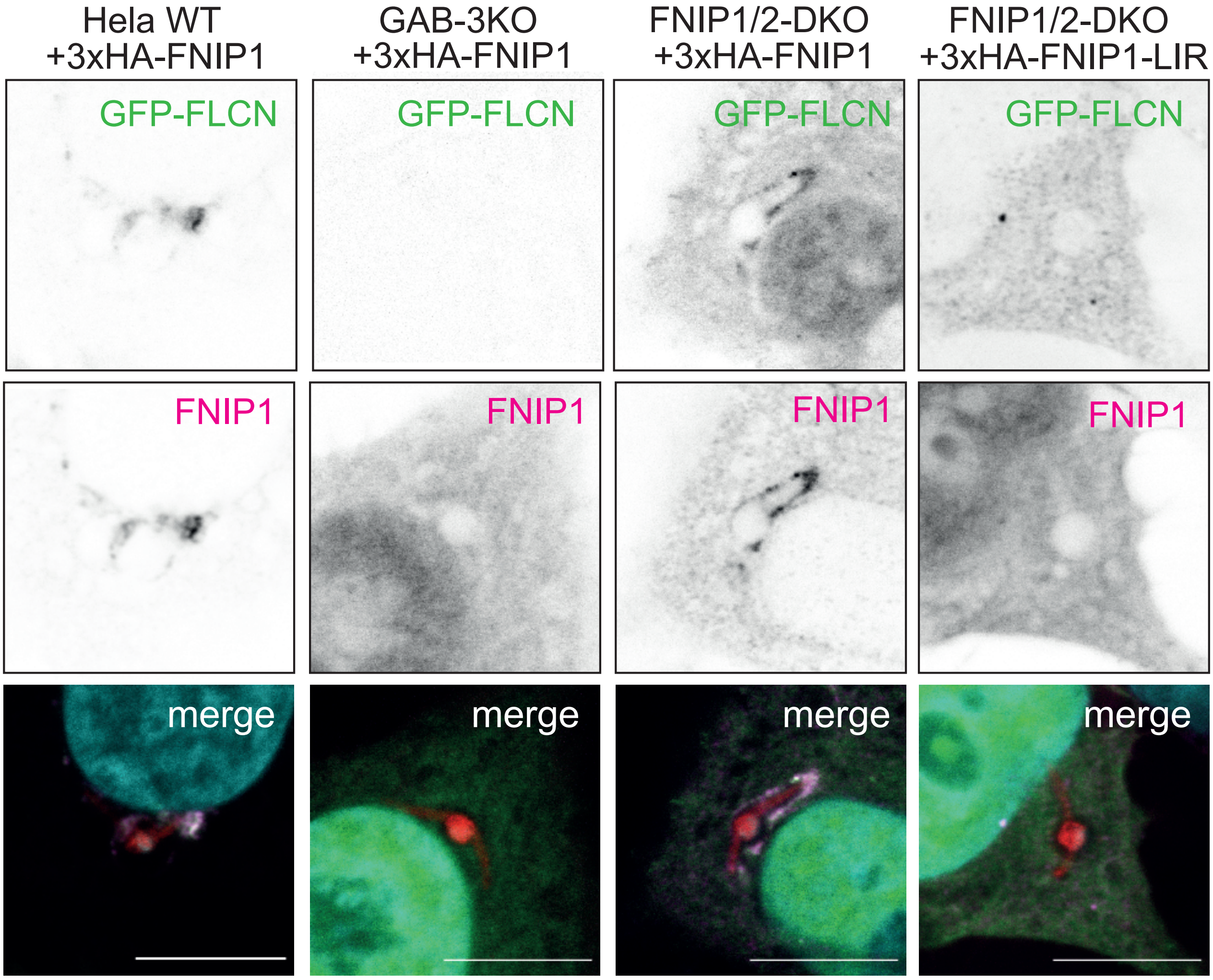

Figure S6: FLCN localization at *Pb* PVM depends on FNIP1. HeLa WT, GAB-3KO cells both constitutively expressing 3xHA-FNIP1 (magenta) and FNIP1/2-DKO cells expressing either 3xHA-FNIP1 (magenta) or 3xHA-FNIP1-LIR (magenta) were transiently transfected with GFP-FLCN (green). Transfected cells were infected with *Pb*mCh (red) approx. 15 h after transfection. 6 hpi cells were fixed and stained with anti-GFP (green) and anti-HA (magenta). DNA was stained with Dapi (cyan). Images were taken with a confocal laser scanning microscope. Scale bar 10 µm. Note: FLCN only can be found at the *P. berghei* PVM together with FNIP1.

Figure S7 Expression of 3xHA-FNIP1, 3xHA-FNIP1-LIR and HA-RagD77L in FNIP1/2-DKO cells

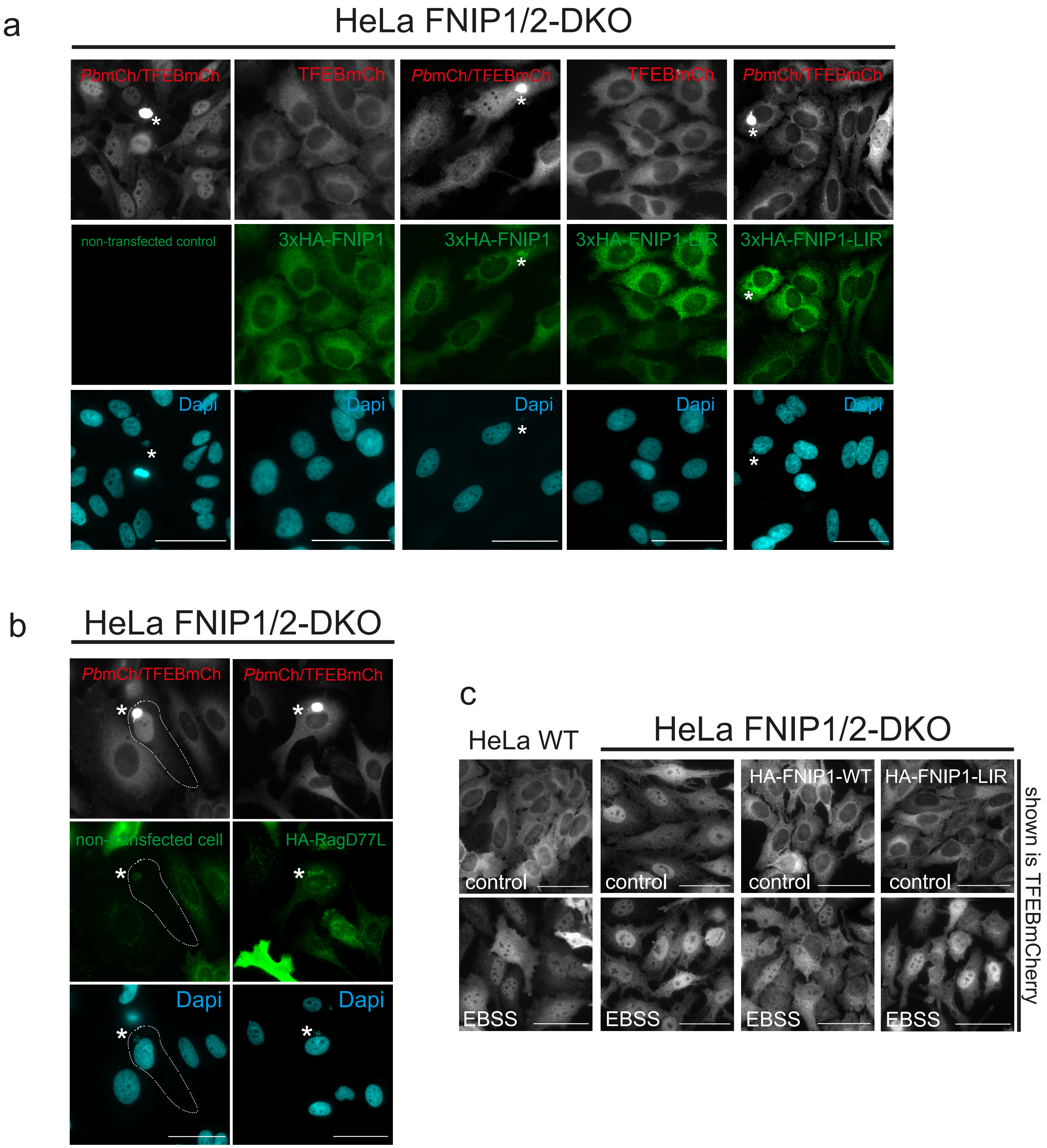

Figure S7: Expression of 3xHA-FNIP1, 3xHA-FNIP1-LIR and HA-RagD77L in FNIP1/2-DKO cells. IFAs used in quantifications of Figures 4d and 4e. **a** TFEB activation in *Pb* infected cells depends on *Pb* PVM localization of FNIP1. Visualisation of cell lines used for the experiment shown in Figure 4d. All cell lines constitutively express TFEBmCh (shown in grey). FNIP1-DKO cells additionally constitutively express 3xHA-FNIP1, 3xHA-FNIP1-LIR (both shown in green). Cells were infected with *Pb*mCh (shown in grey) and fixed 24 hpi and stained with anti-RFP and anti-HA antibodies. Pictures were taken with a fluorescence widefield microscope. *Pb*mCh parasites are labeled with a white asterix. Note that TFEB is only activated when FNIP1-WT is sequestered to the *Pb* PVM. The FNIP1-LIR construct does not localise at the parasite's PVM and does therefore not activate TFEB. In non-infected cells expressing each variant of FNIP1, TFEB is localised in the cell cytoplasm. Scale bar 50 µm. **b** Visualisation of cells used in the experiment shown in Figure 4d. HeLa FNIP1/2-DKO cells constitutively expressing TFEBmCh (shown in grey) were transiently transfected with constitutive active HA-RagD77L (shown in green). Transfected cells were infected with *Pb*mCh (shown in grey) and fixed 24 hpi and stained with anti-RFP and anti-HA antibodies. Pictures were taken with a fluorescence widefield microscope. *Pb*mCh parasites are labeled with a white asterix. Note that in HA-RagD77L transfected, *Pb*mCh infected cells TFEB is localised in the host cell cytoplasm, whereas TFEB localises to the host cell nucleus in a non-transfected, infected cell (left panel, surrounded with a white line). Scale bar 50 µm. **c** Visualisation of starvation experiment shown in Figure 4e. Same cell lines as described in b. Cells were left untreated (upper panels) or starved for 2 h in EBSS (lower panels). Shown is TFEBmCh in grey. Cells were fixed and stained with anti-RFP antibodies. Pictures were taken with a fluorescence widefield microscope. Scale bar 50 µm. Note that all cell lines can activate TFEB upon starvation.

Supplementary Figure S8

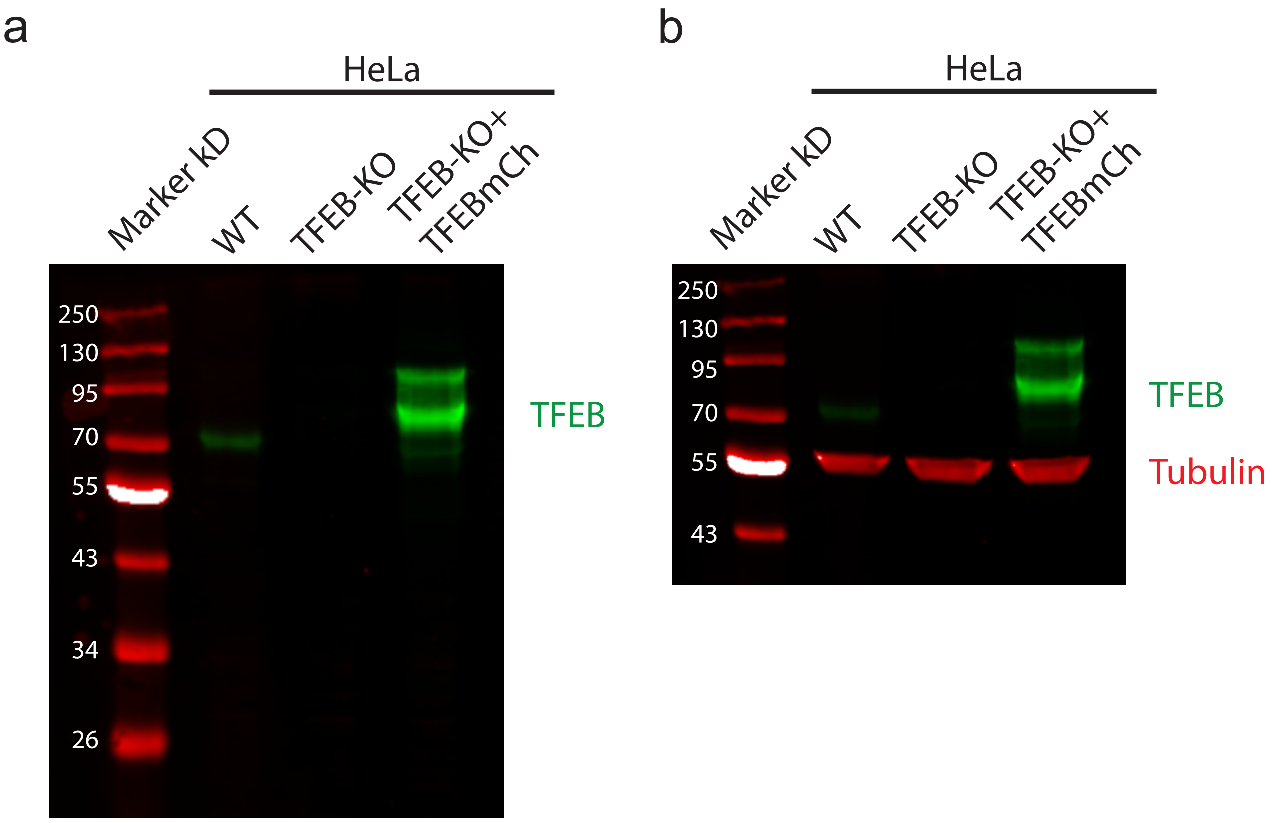

Figure S8: Non croped images of the Western blot shown in Figure 5c. Whole protein lysates were separated on a 12% acrylamide gel, transferred onto a nitrocellulose membrane, and probed with an anti-TFEB antibody here shown in green. Alpha-tubulin was detected as a loading control here shown in red. PageRuler™ Prestained NIR Protein Ladder was used as a size marker, shown in red. Note that in line 3, the TFEBmCh fusion protein expressed in the TFEB-KO cells is larger than the endogenously expressed TFEB due to the mCherry fusion partner.
