# Supplementary material for: Plasmodium berghei liver stage parasites exploit host GABARAP proteins for TFEB activation
Source: Commun Biol. 2024 Nov 21;7:1554. doi: 10.1038/s42003-024-07242-x (PMC11582615; doi:10.1038/s42003-024-07242-x)
Supplement: Supplementary file 3 — Description of Additional Supplementary Materials [file 42003_2024_7242_MOESM3_ESM.pdf]

Description of Additional Supplementary Files

File name: Supplementary Data 1

Description: Contains the numerical source data of all graphs presented in the main figures and the supplementary figures
